# Research trends in nutritional interventions for stroke: a bibliometric analysis and literature review

**DOI:** 10.3389/fnut.2024.1489222

**Published:** 2024-10-16

**Authors:** Yipeng Xie, Yuan Xiong, Mengyue Sun, Yan Zhao, Miao Wu

**Affiliations:** ^1^School of Acupuncture-Moxibustion and Orthopedics, Hubei University of Chinese Medicine, Wuhan, China; ^2^Department of Tuina and Rehabilitation Medicine, Hubei Provincial Hospital of Traditional Chinese Medicine, Wuhan, China; ^3^Department of Tuina and Rehabilitation Medicine, Affiliated Hospital of Hubei University of Chinese Medicine, Wuhan, China; ^4^Department of Tuina and Rehabilitation Medicine, Hubei Institute of Traditional Chinese Medicine, Wuhan, China; ^5^First Clinical Medical College, Hubei University of Chinese Medicine, Wuhan, China

**Keywords:** stroke, nutrition, bibliometry, global trends, analysis of references

## Abstract

**Background:**

Over the past 23 years, there has been a thorough analysis of literature concerning nutritional interventions, nutrients, and feeding approaches related to stroke. Furthermore, a scientific knowledge map was established, elucidating the current state of research, examining its development and trends, and offering new research viewpoints for the future. This study aimed to investigate global and emerging research trends in nutritional interventions for stroke from 2000 to 2023 through bibliometric analysis.

**Methods:**

A bibliometric analysis of literature from the Core Collection of Scientific Networks for the years 2000–2022 was conducted. CiteSpace, VOSviewer, and bibliometric graphical software were used to identify major contributors to publications, including authors, countries, institutions, journals, references, and keywords.

**Results:**

The bibliometric analysis yielded a total of 464 publications. This is a gradually increasing number in terms of the number of publications during the study period. China had the highest number of publications. Clinical Nutrition” was the journal with the highest number of relevant publications, and the most commonly used keywords were “stroke,” “nutrition” and “malnutrition.”

**Conclusion:**

These analyses reveal research trends in nutritional therapy for stroke from 2000 to 2023 and point to prospective research frontiers. This study provides a deeper understanding of what nutritional treatment of stroke entails and provides guidance and support for future research in this area.

## 1 Introduction

Stroke is a condition resulting from the sudden rupture of brain vessels or a blockage that prevents blood flow to the brain, leading to brain tissue damage. It is the second leading cause of death and disability worldwide (1). Patients’ physical functions and quality of life are severely impacted by post-stroke disabilities, imposing a heavy burden on families and society. Following treatment, improvements in cerebral ischemia or hemorrhage are observed in stroke patients, and symptoms of brain tissue damage may also improve. Nevertheless, many patients face considerable challenges with nutritional supplementation, complicating even basic eating and hindering their ability to undergo rehabilitation due to their weakened condition. Approximately one-fifth of stroke patients are malnourished at the time of hospital admission, with malnutrition prevalence fluctuating between 6.1 and 62% (2, 3). And studies suggest that providing appropriate nutritional support after a stroke can effectively reduce disabilities and improve quality of life. Current research indicates that nutritional intervention post-stroke has become an indispensable part of clinical medical practice. However, it is worth noting that nutritional science covers a broad range, thus necessitating a thorough evaluation and review of scientific publications in the field of post-stroke nutrition, aimed at enhancing our understanding of current clinical directions. Although there are many reviews on post-stroke nutrition, they often lack targeted visual data support and heavily rely on researchers’ subjective understanding of the disciplinary framework. Therefore, these reviews exhibit certain levels of heterogeneity and subjectivity, impeding our comprehensive analysis and determination of the research status, identification of hotspots, and recognition of the forefront. This study utilizes bibliometric analysis methods to perform a visual analysis of publications in post-stroke nutrition over the last 23 years, covering involved countries and institutions, keywords, references, themes, disciplines, and sources from journals. This comprehensive analysis aims to determine the distribution of current research findings, identify major contributors, precisely locate research hotspots, assess the status quo, and explore the forefront of the field, thereby establishing a systematic and comprehensive knowledge base. This framework provides convenience for researchers in the field, enabling them to explore its breadth and serving as a valuable resource for newcomers, facilitating their further research (4).

Bibliometrics is an interdisciplinary field of mathematics and statistics that describes and analyses the dynamics and progress of a specific discipline or research area, and is widely used to identify research hotspots and trends, helping us understand the knowledge base and advancements at the forefront of a specific field. Bibliometric analysis has increasingly been applied to stroke-related research, including studies on post-stroke depression (5), stroke and diabetes (6), and post-stroke dysphagia (7). With the further strengthening of international communication and collaboration, researchers around the world are placing greater emphasis on nutritional research related to stroke. This study employs bibliometric methods to conduct a detailed and systematic analysis of the current state and trends in stroke-related nutritional research. We review the classification of research related to stroke nutrition, research prospects, popular areas of study, and development trends.

## 2 Data analysis and results

### 2.1 Data sources and search strategy

We retrieved the Science Citation Index Expanded (SCI-Expanded) from the Web of Science Core Collection (WoSCC) database and completed all searches by February 4, 2024, to avoid biases due to database updates. The Institute for Scientific Information (ISI) Web of Science database is the largest and most comprehensive academic information resource globally, which includes a variety of influential core journals across fields such as natural and social sciences. The retrieval strategy is as follows: TS = (“nutrition” OR “nutritional therap*” OR “nutritional assessment” OR “nutritional supplement*” OR “nutritional intervention*”) AND TS = (“stroke” OR “apoplexy” OR “cerebrovascular accident” OR “cerebral hemorrhage” OR “hematencephalon” OR “encephalorrhagia” OR “cerebral ischemia”) AND DOP = (2000-01-01/2024-02-04) AND LA = (English) and Article (Document Types).

In this study, the keywords “nutritional therap” and “nutritional therapies” were standardized to “nutritional therap.” “Nutrition” represents most research related to diet, nutrition, and nutritional therapy; “Nutritional therap” uses the wildcard “*” to indicate “nutritional therapy” and “nutritional therapies,” emphasizing research on nutritional treatment methods; “Nutritional assessment” refers to the evaluation of nutritional status, involving literature that assesses patients’ nutritional conditions; “Nutritional supplement” uses the wildcard “*,” encompassing both “nutritional supplement” and “nutritional supplements,” referring to studies related to nutritional supplementation; “Nutritional intervention” focuses on nutritional interventions aimed at improving health through specific nutritional measures, such as dietary adjustments. Potentially excluded synonyms include “diet therapy” and “nutritional support.” “Stroke” is the most common term for cerebrovascular accidents, “Apoplexy” may appear in older literature, “Cerebrovascular accident” is more commonly found in clinical literature, “Cerebral hemorrhage” specifically refers to hemorrhagic stroke, “Hematencephalon” and “encephalorrhagia” may appear in advanced medical literature, “Cerebral ischemia” refers to ischemic stroke, while “brain attack,” a newer term, may be excluded due to its lower frequency of use.

To ensure the relevance and quality of the included literature, we have adopted the following inclusion and exclusion criteria: (1) Two members of the team independently evaluated the papers, excluding those that were inconsistent with the topic. (2) The selected documents, institutions, and countries were standardized to prevent variations in names from affecting the results. For example, England, Scotland, Wales, and Northern Ireland were combined into the UK, and Taiwan was merged with China. (3) The selected research topics must align with journal articles related to post-stroke nutrition research, such as pharmacological studies of specific nutrients, nutritional interventions and management studies for post-stroke rehabilitation patients, and studies on nutritional screening and assessment. (4) Manual filtering was employed to review all titles and abstracts of relevant articles, to enhance the accuracy and scientific rigor of the search data by excluding literature related to cardiovascular diseases and other cerebrovascular diseases, as these two categories of diseases are not within the scope of this study. (5) The literature must include sufficient data and information, such as country, research institution, author details, etc., to facilitate visual analysis.

**Figure 1:**
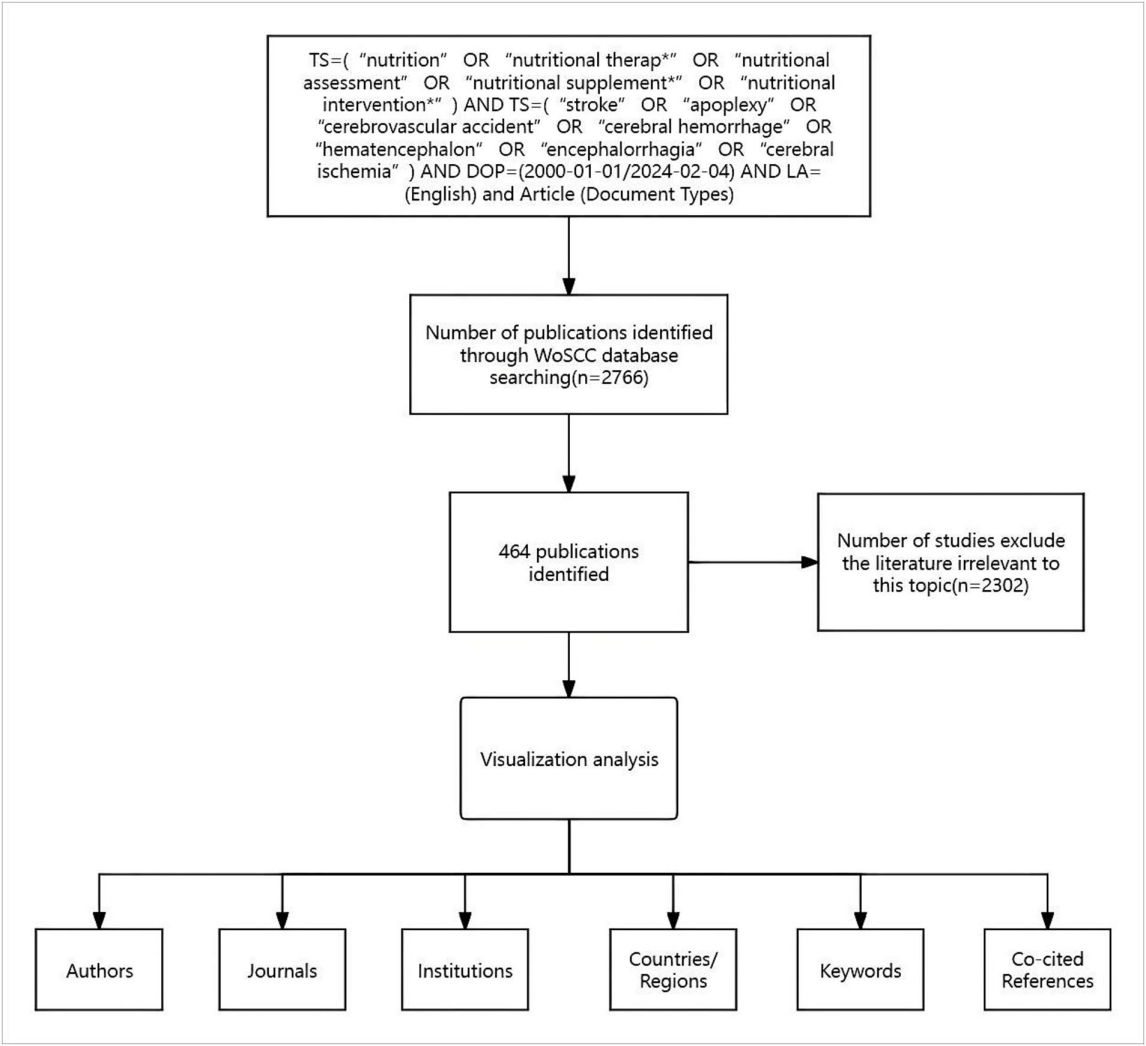
Flowchart of research process.

Ultimately, 2,302 articles that were irrelevant to post-stroke nutrition were excluded, resulting in the inclusion of 464 articles (Figure 1).

### 2.2 Bibliometric tools

The following software tools were used in this study: VOSviewer (version 1.6.18; Leiden University, Netherlands), CiteSpace (version 6.2.R4; Chaomei Chen, Drexel University, Philadelphia, PA, United States), Microsoft Excel (Redmond 2019; Washington State, United States), the Bibliometrix package based on R (version 4.1.3), and Scimago Graphica (Beta 1.0.36). VOSviewer excels at constructing visual networks based on co-occurrence relationships, clearly displaying the co-occurrences of authors, institutions, and keywords, thus aiding in the discovery of academic networks and research hotspots within the field (8). CiteSpace is adept at analyzing research frontiers, co-citation analysis, and burst detection, particularly in identifying hotspots and evolutionary trends within a specific research domain (9). These tools are used for data analysis, with results exported as tables summarizing key bibliometric indicators, including the number of publications distributed by year, total citations, average citations, titles, countries and institutions, authors, journals, keywords, and references. Co-occurrence maps of countries/regions were generated using VOSviewer and Scimago Graphica, while the co-occurrence and collaboration networks among institutions were visualized using VOSviewer. In the VOSviewer map, nodes represent collaboration or co-occurrence in the literature, and the thickness of the lines is positively correlated with connection strength. In modular visualizations, different colors are used to distinguish clusters, while in overlay visualizations, node colors indicate the average publication year, with blue representing earlier research and orange indicating more recent work. CiteSpace is used to generate burst detection maps for keywords and identify key references, with dark nodes corresponding to earlier publications and light nodes representing more recent publications. Additionally, in the burst detection module, keywords and references are sorted based on the starting year of the burst period. The Bibliometrix package based on R is used to create three-field plots to illustrate the relationships between co-cited countries, institutions, and keywords. Scimago Graphica visualizes the breadth and depth of collaboration among countries through nodes and connecting lines.

### 2.3 Scientific production

**Figure 2:**
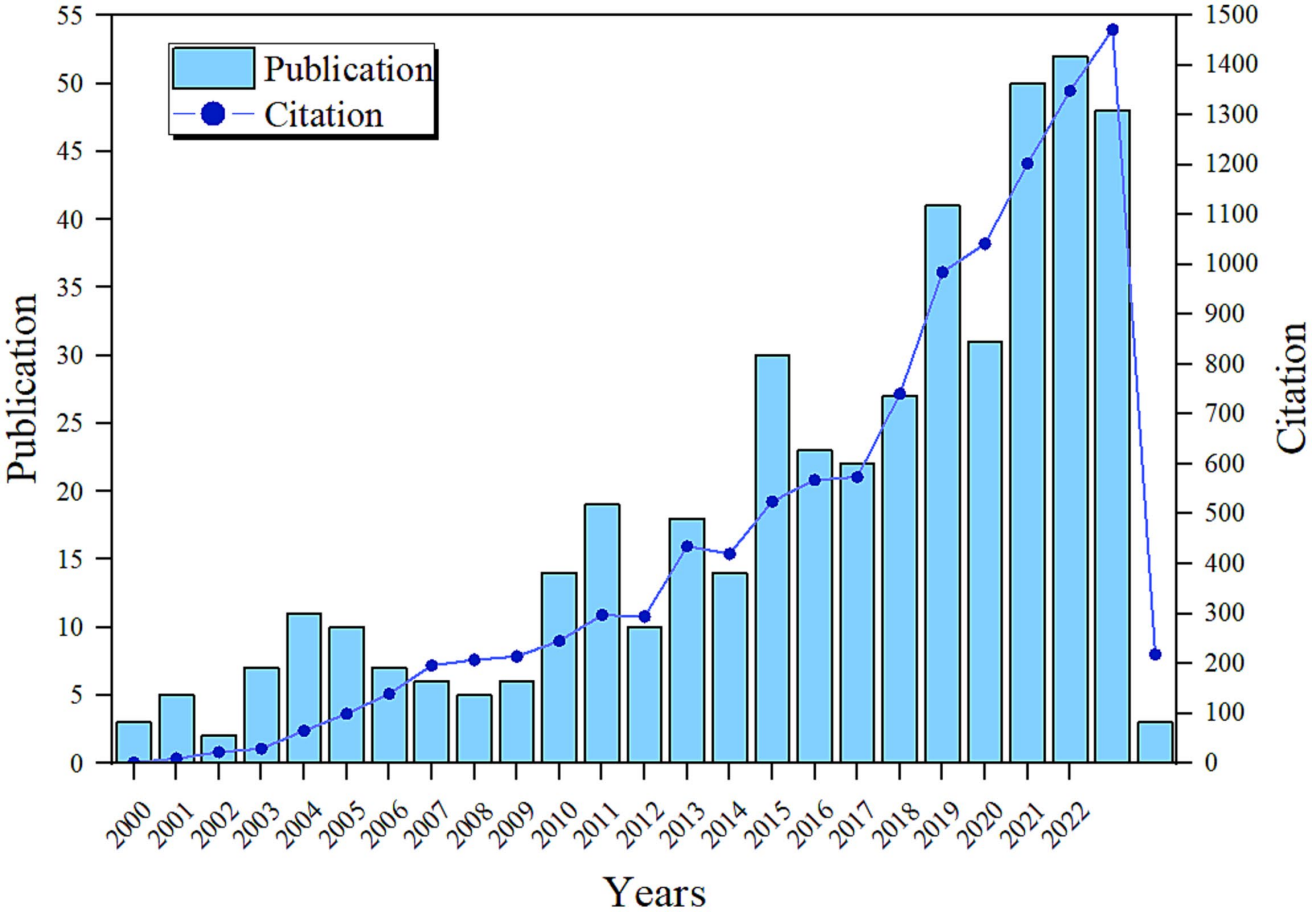
Annual and cumulative output and publication trends of global post-stroke nutrition research publications from 2000 to 2023.

Our search revealed that from January 1, 2000, to January 12, 2024, there were 464 papers worldwide related to post-stroke nutrition (Figure 2). The volume of literature has steadily increased since 2000, with a significant rise after 2010, reflecting a growing focus and research interest in this area. Observing the changes in citation count, we found that citations increased with the volume of publications, and at a faster rate, indicating a gradual increase in the influence and recognition of published literature on subsequent research. From the trends in publication and citation growth, it is evident that research on post-stroke nutritional intervention has become a hot topic in academia and clinical practice, with both depth and breadth of research continually expanding, including basic and clinical studies. The number of papers exceeded 30 in 2015, declined in 2020, surpassed 50 in 2021, and peaked at 52 in 2022. The publication of a high-quality paper in 2009 titled “Mediterranean Diet and Incidence of and Mortality From Coronary Heart Disease and Stroke in Women” has significant academic value and has attracted widespread attention from researchers, leading to a subsequent surge in research on dietary patterns (10–12). More scholars have begun to explore its applicability in different populations and conditions (13, 14), including the mechanisms of specific components in the Mediterranean diet, such as olive oil, nuts, and fish (15, 16). Furthermore, studies combining nutrition and neuroscience after 2010 (17, 18), the exploration of pre-stroke nutrition (19), the development of molecular nutritional research (16), and the expansion of dietary nutrient supplementation have all contributed to the increase in publication volume in this field (21–24). The nutritional management guidelines for neurological diseases published by ESPEN in 2018 (25) have guided clinical practice and further stimulated the increase in publication volume. In 2020, during the COVID-19 pandemic, unprecedented efforts were being made to develop COVID-19 vaccines, and research resources shifted towards pandemic-related studies, as evidenced by the cancellation of an originally planned physical consensus conference due to the COVID-19 pandemic (26). Consequently, the number of publications related to stroke nutrition declined. To this day, researchers remain highly enthusiastic about this field, continually working together to expand the breadth and depth of the field, resulting in a new peak in publication volume.

### 2.4 Countries/regions

**Figure 3:**
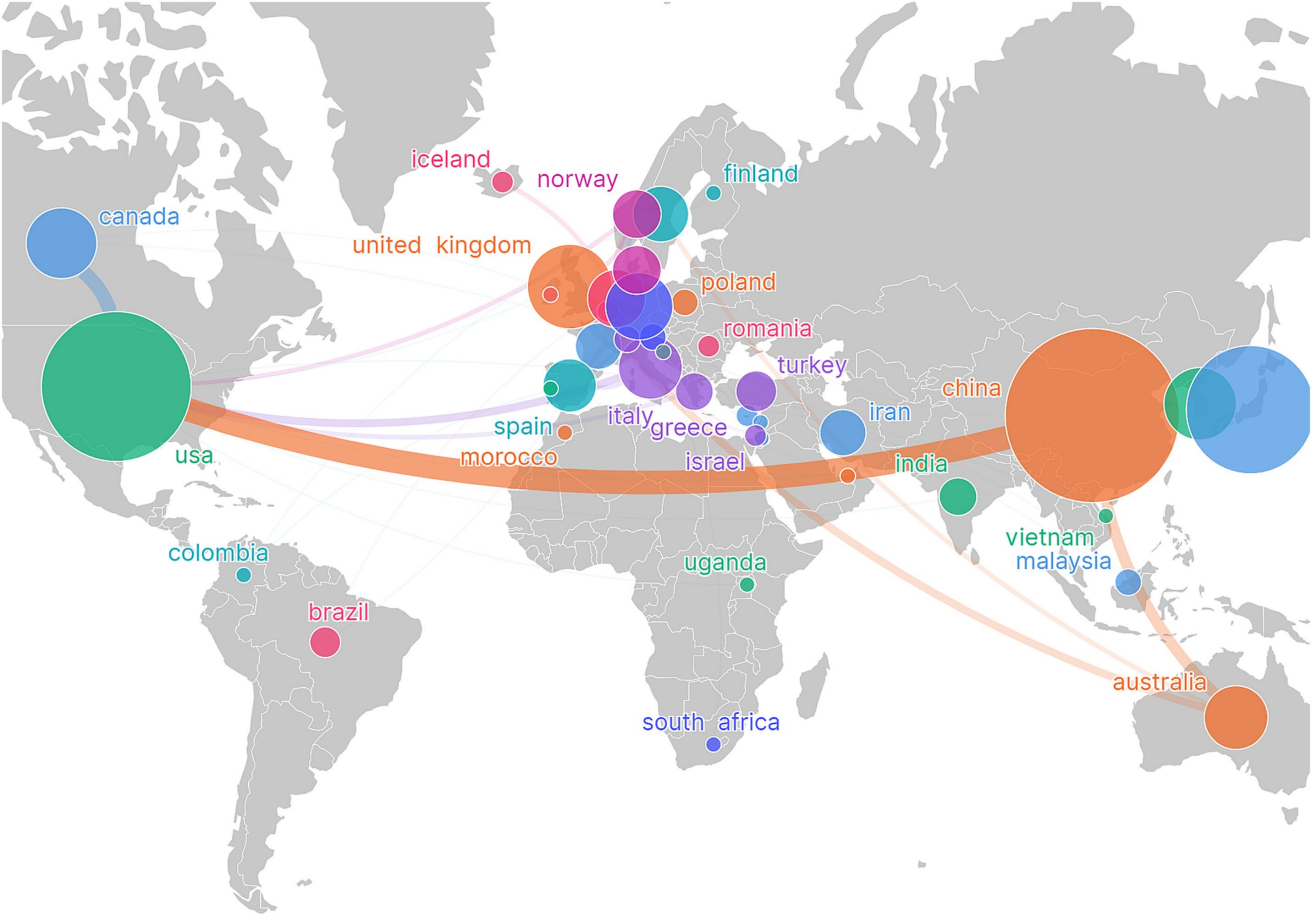
Visualization of country/region network, showing global research collaboration. The size of the nodes is proportional to the publication volume of the countries, while the thickness of the connecting lines indicates the strength of collaboration between the countries. Larger nodes represent a higher publication volume, and thicker lines indicate a greater degree of collaboration.

Table 1 shows the global collaboration and influence distribution in the field of post-stroke nutritional intervention, with numerous intercontinental connections indicating international scientific collaboration. The USA and China likely have a high publication volume or frequent collaborations, suggesting their dominant role in research cooperation in this field. The top 10 countries/regions by number of published papers are listed, with China (*n* = 127), the USA (*n* = 94), and Japan (*n* = 69) leading the ranks. In terms of the H-index, China (*n* = 127), the USA (*n* = 94), and Japan (*n* = 69) rank the highest. Figure 3 demonstrates the close cooperation between China and the USA. Table 1 shows the annual changes in the number of papers published by the top 10 countries, revealing that most reached their peak in publication numbers in 2021, 2022, and 2023.

**Table 1:** Major research countries.

Rank	Country	Document	Citation	Citation/ document
1	China	127	1,394	17
2	USA	94	4,418	31
3	Japan	69	1,279	0
4	United Kingdom	30	1862	24
5	South Korea	22	157	1
6	Canada	21	649	8
7	Germany	19	769	24
8	Australia	17	553	11
8	Italy	17	511	23
10	Netherlands	14	468	29

### 2.5 Organizations

**Figure 4:**
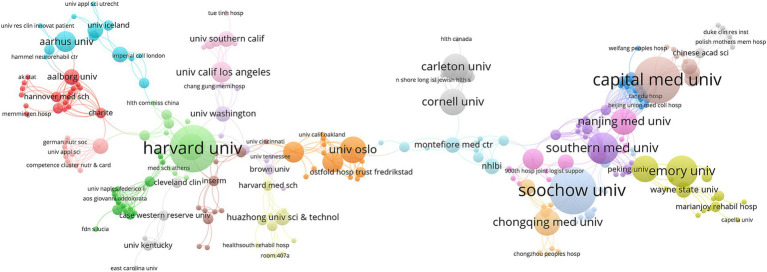
Network visualization map of relevant institutions from 2001 to 2024. The size of the nodes is proportional to the publication volume of the institutions, while the thickness of the connecting lines indicates the strength of collaboration between the institutions. Larger nodes represent a higher publication volume, and thicker lines indicate a greater degree of collaboration.

Table 2 lists the top 10 institutions by number of published papers, with Harvard University leading with 14 papers and a high citation rate of 89.07, demonstrating its central position in this field’s research. A higher H-index (11) further indicates that its research is influential and frequently cited. Nagasaki Rehabil Hospital follows with 11 papers. Although it has fewer publications than Harvard University, its high citation impact (H = 10) further demonstrates the groundbreaking and highly relevant nature of its research. Capital Medical University (H = 4) and Soochow University China (H = 5) are tied for third place with 9 papers each, indicating that these institutions are increasingly playing a significant role in global collaborative research (Figure 4).

**Table 2:** Major research institutions.

Rank	Institution (country)	Document	Citation	Citation/ document	H-index
1	Harvard University	14	1,247	89.07	11
2	Nagasaki Rehabilitation Hospital	11	344	31.27	10
3	Capital Medical University	9	57	6.33	4
3	Soochow University China	9	189	21	5
5	University of London	8	242	30.25	6
6	Aarhus University	7	206	29.43	4
6	Brigham Women’s Hospital	7	981	140.14	7
6	Johns Hopkins University	7	252	36	7
6	Karolinska Institutet	7	171	24.43	5
6	Sichuan University	7	91	13	5
6	Southern Medical University China	7	66	9.43	3
6	Wenzhou Medical University	7	133	19	6
6	Western University of Western Ontario	7	262	37.43	7

### 2.6 Journals

In the top 10 journals (Table 3), “Clinical Nutrition” leads with 29 publications, a total of 1,018 citations, averaging 35.1 citations per article, an impact factor of 6.3, and an H-index of 17, demonstrating its authority and research quality in the field of clinical nutrition. “Nutrients” (H = 9) follows closely with 23 publications, displaying high research activity and strong academic influence. “Journal of Stroke & Cerebrovascular Diseases” (H = 11) and “Stroke” (H = 20) each with 23 publications rank third. “Stroke” has the highest citation count at 1647, significantly higher than other journals of the same rank, highlighting its importance and depth of research in the field of stroke. “European Journal of Clinical Nutrition” and “Nutrition” also demonstrate their importance in the field, although the number of publications is smaller, the average citations per publication and impact factors are notably high.

**Table 3:** Top 10 major journals by number of publications.

Rank	Journal	Document	Total citation	Citation/ document	IF2023	H-index
1	Clinical Nutrition	29	1,018	35.1	6.3	17
2	Nutrients	25	257	10.28	5.9	9
3	Journal of Stroke Cerebrovascular Diseases	23	385	16.74	2.5	11
3	Stroke	23	1,647	71.61	8.4	20
5	Nutrition Metabolism and Cardiovascular Diseases	17	202	11.88	3.9	7
6	European Journal of Clinical Nutrition	13	426	32.77	4.7	10
7	Nutrition	11	450	40.91	4.4	8
8	Frontiers in Neurology	9	46	5.11	3.4	3
8	Journal of Parenteral and Enteral Nutrition	9	96	10.67	3.4	5
10	Asia Pacific Journal of Clinical Nutrition	8	46	5.75	1.3	4
10	Nutritional Neuroscience	8	92	11.5	3.6	6

### 2.7 Authors

Data from Table 4 shows the top ten authors by publication volume are Nishioka Shinta (*n* = 11), Wakabayashi Hidetaka (*n* = 9), Boeing Heiner (*n* = 6), Paterson Phyllis G. (*n* = 5), Maruyama Hirofumi (*n* = 4), Aoki Shiro (*n* = 4), Jadavji Nafisa (*n* = 4), Nezu Tomohisa (*n* = 4), Weikert Cornelia (*n* = 4), Nozoe Masafumi (*n* = 4), Verschuren W.M.Monique (*n* = 4), Boer Jolanda M. A. (*n* = 4), Maeda Keisuke (*n* = 4), Chirlaque Maria-Dolores (*n* = 4), Domen Kazuhisa (*n* = 4), and Kayashita Jun (*n* = 4). The top five authors by H-index are Nishioka Shinta (10), Wakabayashi Hidetaka (8), Boeing Heiner (5), Paterson Phyllis G. (4), and Maruyama Hirofumi (3). Nishioka Shinta has the highest number of publications, with an average of 31.18 citations per paper and an H-index of 10, highlighting his significant contributions and the broad recognition of his research. Nishioka Shinta, as a leading contributor in this field, primarily focuses on swallowing disorders and sarcopenia following stroke. He and his research team have thoroughly explored how nutritional interventions can improve swallowing function in stroke patients (27), revealing the relationship between malnutrition and sarcopenia (28, 29), which has significant implications for developing guidelines for malnutrition screening and intervention. Wakabayashi Hidetaka focuses on investigating how nutritional interventions can enhance rehabilitation outcomes in stroke patients. For example, he examines the relationship between sarcopenia in stroke rehabilitation patients and activities of daily living and swallowing (30), the association between low hemoglobin levels and poor rehabilitation outcomes (31, 32), and the relationship between nutritional improvement and energy intake and functional recovery in post-stroke patients (33, 34). His research provides important guidance for clinical nutritional rehabilitation, particularly in reducing complications and improving quality of life. Boeing Heiner primarily investigates the relationship between dietary habits and stroke risk, providing empirical support for dietary patterns (such as the Mediterranean diet) in stroke prevention (35), exploring the risk relationships between different food groups and stroke, and emphasizing the importance of proper nutrition (36).

**Table 4:** Author publication volume and H-index.

Rank	Author	Publications	Citations	Average citations/publication	H-index
1	Nishioka Shinta	11	343	31.18	10
2	Wakabayashi Hidetaka	9	281	31.22	8
3	Boeing Heiner	6	207	34.50	5
4	Paterson Phyllis G.	5	81	16.20	4
5	Maruyama Hirofumi	4	82	20.50	3
5	Aoki Shiro	4	82	20.50	3
5	Jadavji Nafisa	4	32	8.00	2
5	Nezu Tomohisa	4	82	20.50	3
5	Weikert Cornelia	4	136	34.00	4
5	Nozoe, Masafumi	4	15	3.75	1
5	Verschuren W.M.Monique	4	67	16.75	3
5	Boer, Jolanda M. A.	4	19	4.75	3
5	Maeda, Keisuke	4	161	40.25	4
5	Chirlaque Maria-Dolores	4	29	7.25	3
5	Domen, Kazuhisa	4	58	14.5	4
5	Kayashita, Jun	4	90	22.5	4

### 2.8 Analysis of references and co-cited references

**Figure 5:**
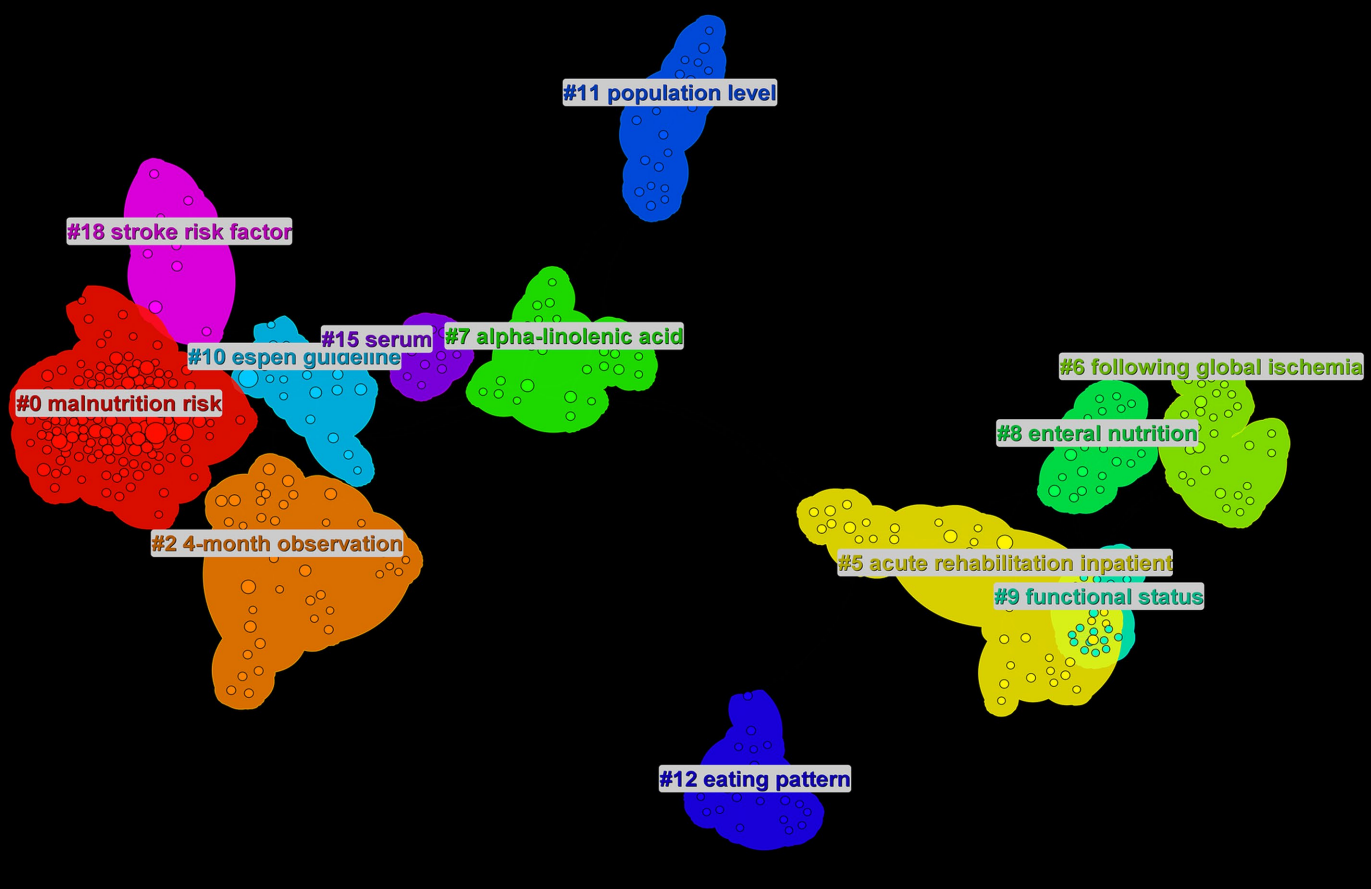
Clustering of references, stroke nutrition related common citation group.

**Figure 6:**
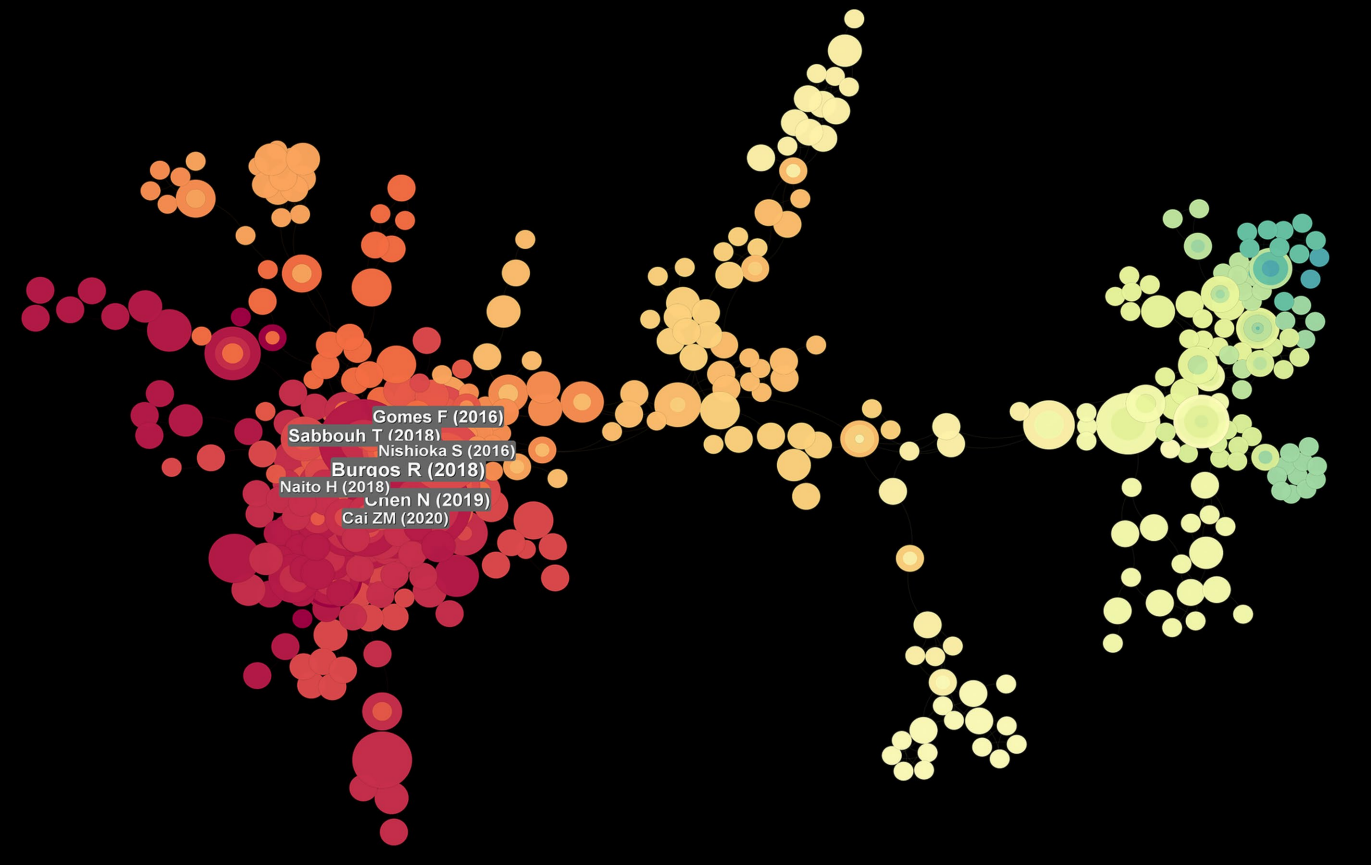
Reference map with co-occurrence visualisation of authors co-citing references.

**Figure 7:**
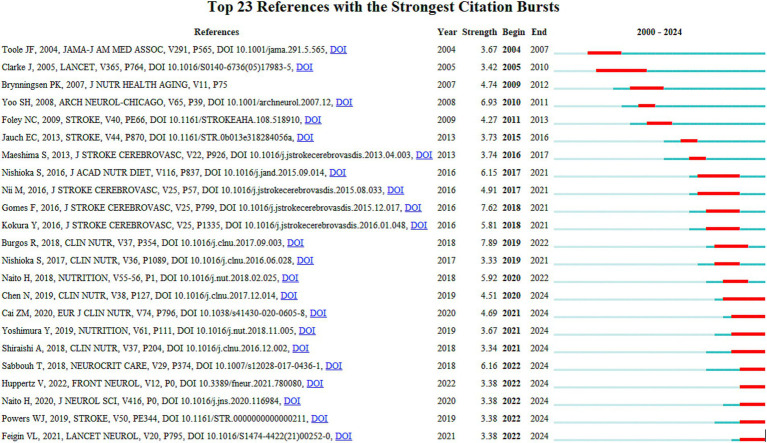
Reference burst map, the “bursts” occurred in the reference network. The top 23 references with the strongest citation bursts are shown here.

Data analysis (Table 5) shows the top 10 co-cited documents include “Effect of Malnutrition after Acute Stroke on Clinical Outcome” at the top with 75 citations, indicating its relevance to clinical outcomes and showing that nutritional interventions in stroke are widely regarded in clinical settings. Using CiteSpace, co-cited references were extracted and then clustered, with each cluster representing a specific research theme and keyword group (Figures 5, 6). Twelve clusters emerged: #0 malnutrition risk; #1 4-month observation; #2 acute rehabilitation inpatient; #3 following global ischemia; #4 alpha-linolenic acid; #5 enteral nutrition; #6 functional status; #7 espen guideline; #8 population level; #9 eating pattern; #10 serum; #11 stroke risk factor. The three clusters with the highest burst are “#0 malnutrition risk,” focusing on the risk of malnutrition; “#1 4-month observation,” concerning nutritional observations over specific months; and “#2 acute rehabilitation inpatient,” highlighting acute rehabilitation patients, indicating that stroke rehabilitation patients are a key research group. A citation burst indicates a significant increase in citations for certain literature within a specific period (Figure 7). The top three references with the highest burst strength are “ESPEN guideline clinical nutrition in neurology” (strength = 7.89), “Risk of Malnutrition Is an Independent Predictor of Mortality, Length of Hospital Stay, and Hospitalization Costs in Stroke Patients” (strength = 7.62), and “Undernutrition as a predictor of poor clinical outcomes in acute ischemic stroke patients” (strength = 6.93). References with high burst strength often encompass more extensive scientific achievements and have a greater impact on subsequent research (Figure 7).

**Table 5:** Ranking of co-cited references.

Rank	Citations	Title	First author	Year	Journal	IF (2023)
1	75	Effect of malnutrition after acute stroke on clinical outcome	Davalos, A	1996	Stroke	8.4
1	74	Poor nutritional status on admission predicts poor outcomes after stroke – Observational data from the FOOD trial	Dennis, M	2003	Stroke	8.4
3	48	Undernutrition as a predictor of poor clinical outcomes in acute ischemic stroke patients	Yoo, SH	2008	Archives of Neurology	2
4	44	Which Reported Estimate of the Prevalence of Malnutrition After Stroke Is Valid?	Foley, NC	2009	Stroke	8.4
5	40	Influence of nutritional status on clinical outcome after acute stroke	Gariballa, SE	1998	American Journal of Clinical Nutrition	7.1
6	34	Impact of premorbid undernutrition on outcome in stroke patients	Davis, JP	2004	Stroke	8.4
7	31	A Review of The Relationship Between Dysphagia and Malnutrition Following Stroke	Foley, NC	2009	Journal of Rehabilitation Medicine	3.5
8	31	Risk of Malnutrition Is an Independent Predictor of Mortality, Length of Hospital Stay, and Hospitalization Costs in Stroke Patients	Gomes, F	2016	Journal of Stroke & Cerebrovascular Diseases	2.5
9	30	Malnutrition in Stroke Patients on The Rehabilitation Service and At Follow-Up – Prevalence and Predictors	Finestone, HM	1995	Archives of Physical Medicine And Rehabilitation	4.3
10	28	ESPEN guideline clinical nutrition in neurology	Burgos, R	2018	Clinical Nutrition	6.3

### 2.9 Keyword analysis

**Figure 8:**
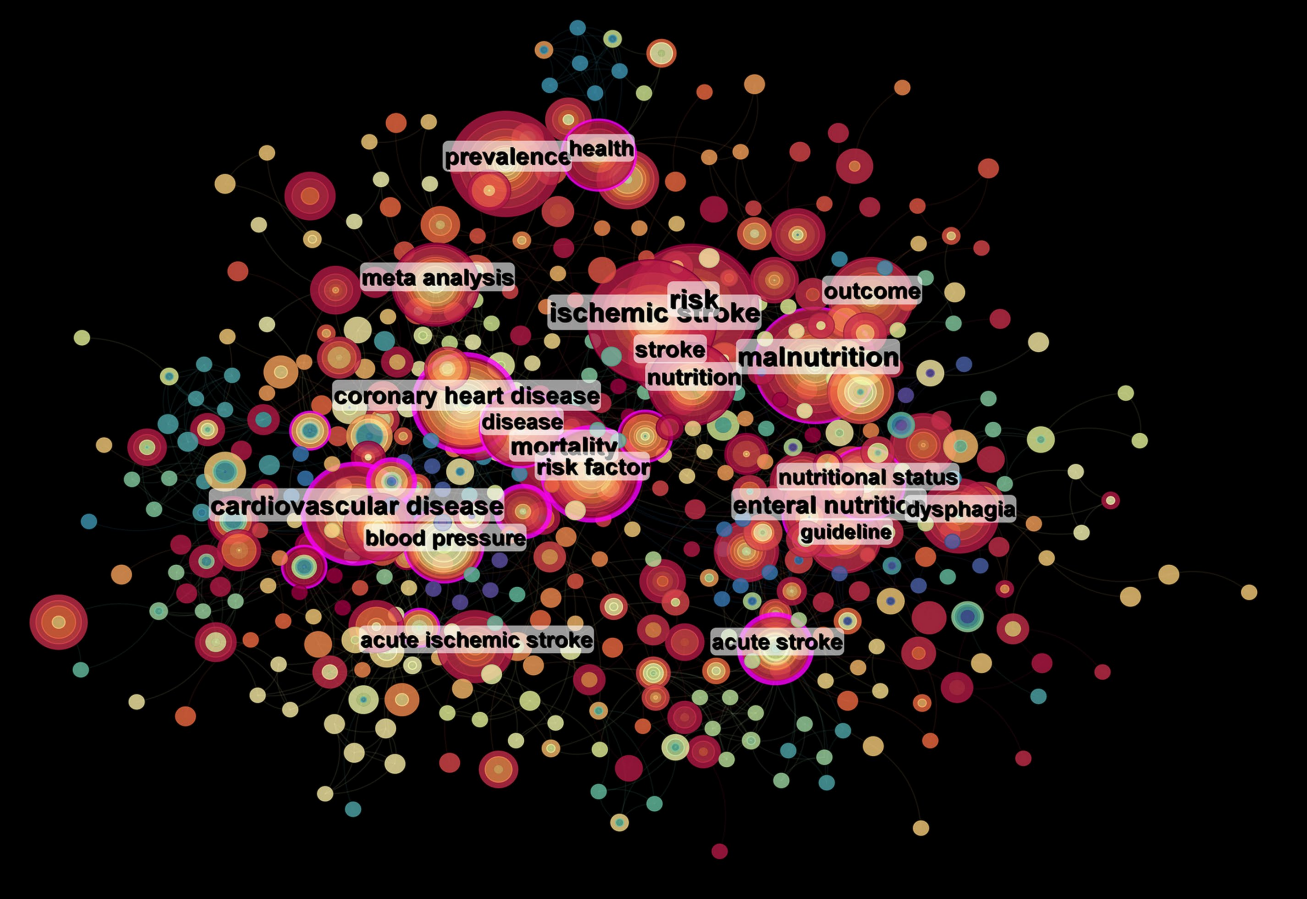
Keyword nodes, in keyword co-occurrence analysis, larger nodes and fonts indicate higher frequencies of keyword occurrences; node colors represent different clustering categories, reflecting various research directions within the field.

**Figure 9:**
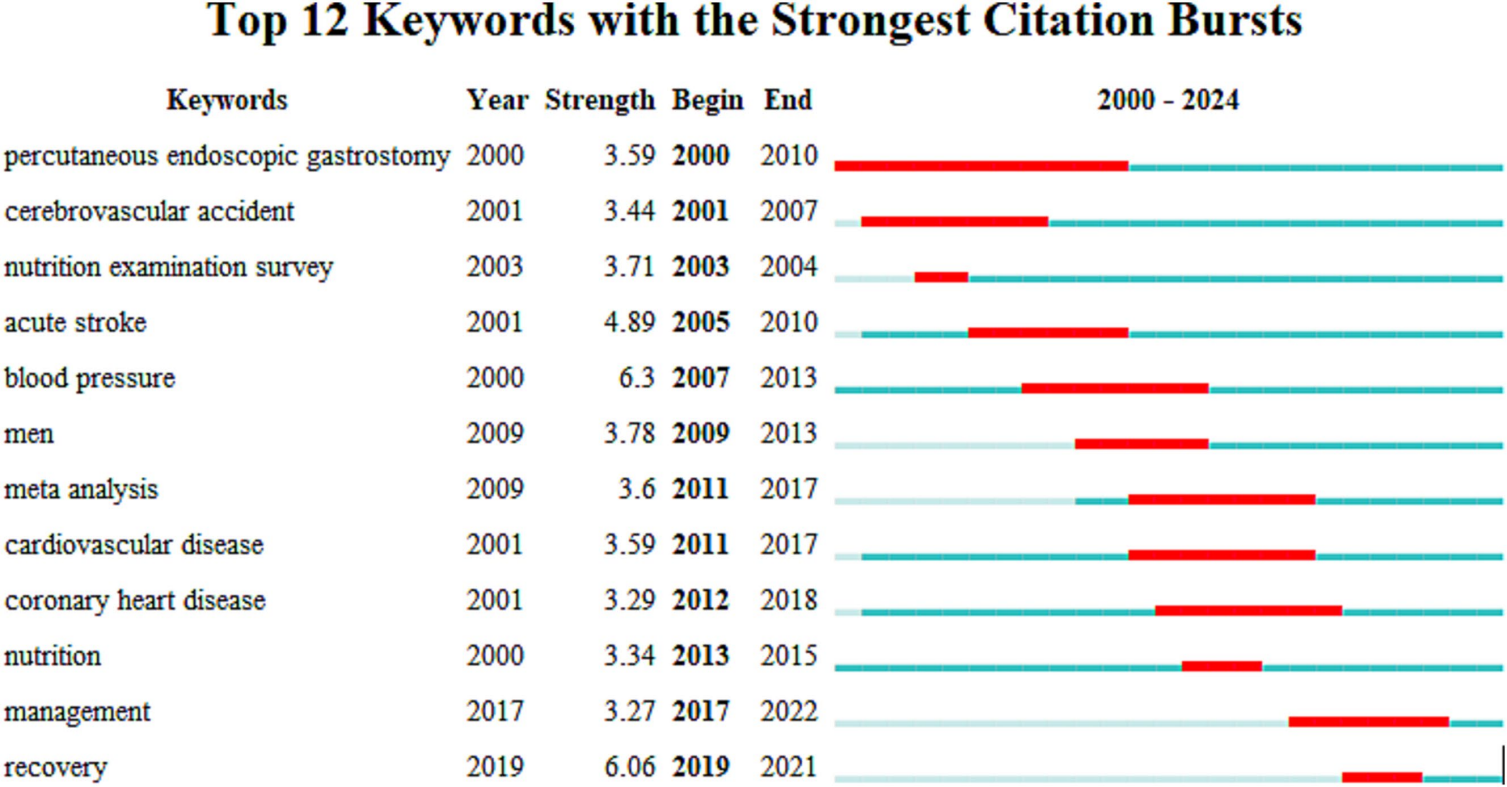
Keyword burst, “burst” occur within the keyword network. The top 12 keywords with the strongest citation bursts are shown in the figure.

**Figure 10:**
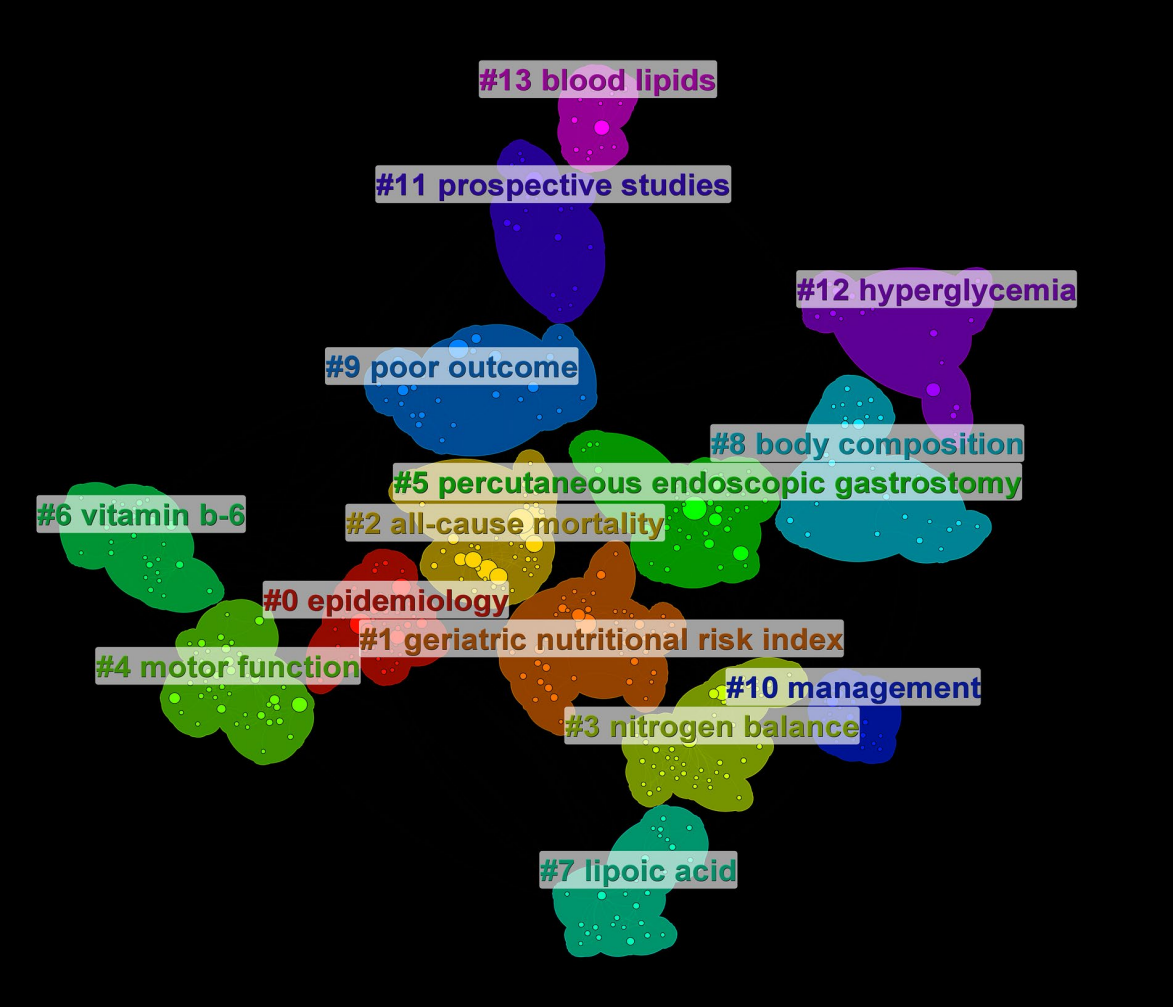
Keyword clustering, different clusters represent different themes, and the size of the clusters indicates research hotspots.

**Figure 11:**
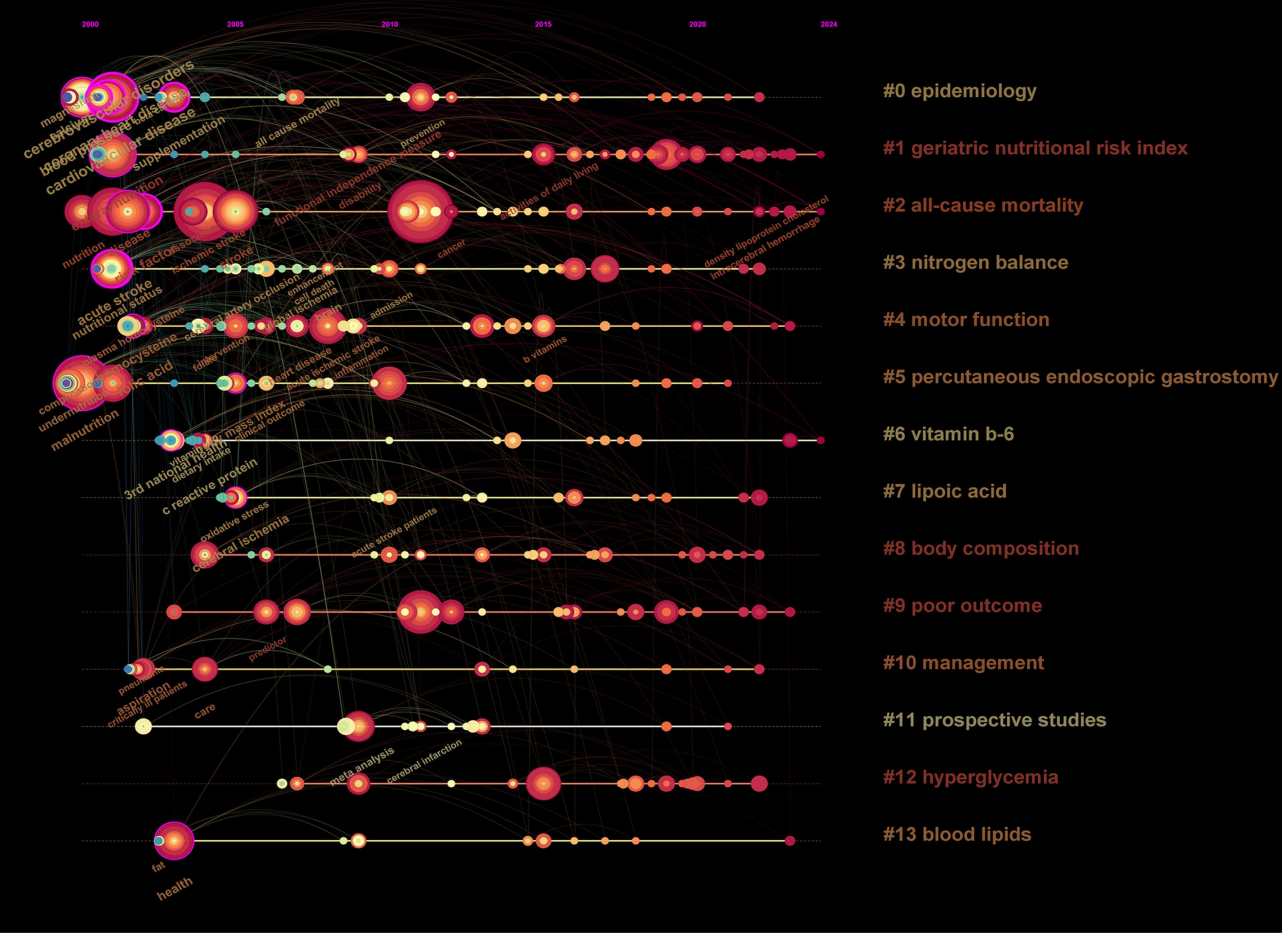
Timeline visualization of keywords, the position of each node represents the year when the keyword first appeared. If the keyword continues to appear in later publications, the frequency of occurrences will accumulate to the year of its first appearance, causing the node to grow larger. The connecting lines indicate the co-occurrence relationship between this keyword and others; thicker lines signify stronger connections.

**Figure 12:**
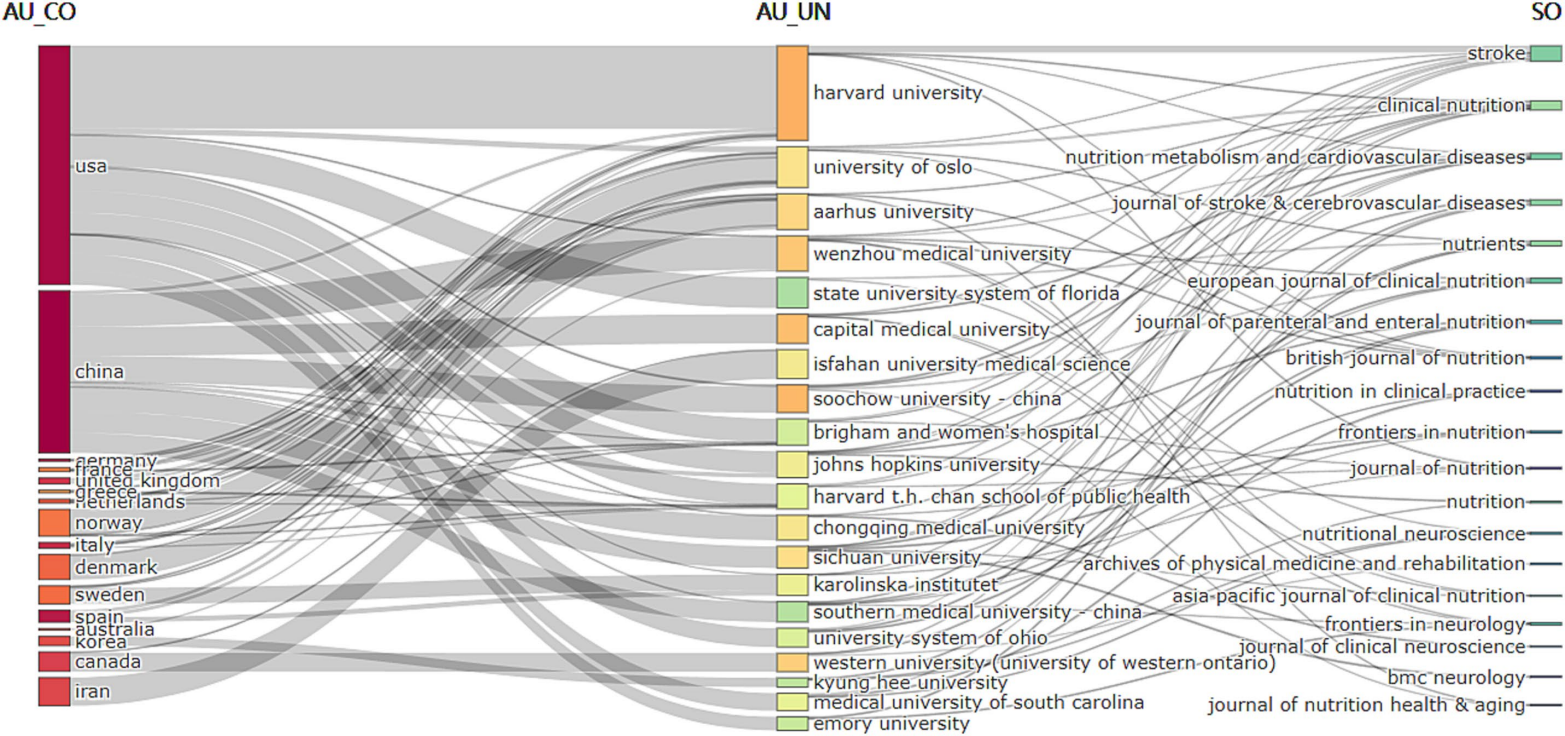
Relationship between the top 20 co-cited countries, institutions, and keywords evolution based on R’s alluvial plot.

The top 20 high-frequency keywords are shown in Table 6 and Figure 8, experiencing a research activity peak from 2000 to 2023. The five most frequent keywords are stroke (*n* = 243), nutrition (*n* = 130), malnutrition (*n* = 103), ischemic stroke (*n* = 88), and risk (*n* = 86), Figure 9 shows the 12 most cited keywords, with their interconnections displayed on a map. Figure 10, a keyword cluster analysis, illustrates the hot topics and key research areas in post-stroke nutrition. Different colored clusters in the diagram represent different research themes, with each numbered label and corresponding keyword displaying the core content of each theme. There are 13 keyword clusters: #0 epidemiology, #1 geriatric nutritional risk index, #2 all-cause mortality, #3 nitrogen balance, #4 motor function, #5 percutaneous endoscopic gastrostomy, #6 vitamin B-6, #7 lipoic acid, #8 body composition, #9 poor outcome, #10 management, #11 prospective studies, #12 hyperglycemia, #13 blood lipids. The keyword timeline visualization (Figure 11) shows the trajectory of research evolution, identifying keywords with sharply increasing frequency in short periods, thus revealing research hotspots and frontier areas. Over time, initial focus areas like epidemiology, geriatric nutritional risk index, all-cause mortality, nitrogen balance, and percutaneous endoscopic gastrostomy shifted towards motor function, poor outcome, and hyperglycemia. The keywords with the highest burst are “blood pressure” (*n* = 6.3), followed by “recovery” (*n* = 6.06) and “acute stroke” (*n* = 4.89). The keyword “percutaneous endoscopic gastrostomy” had the longest burst period, from 2000 to 2010. As of 2024, the most frequently used keywords are “management” and “recovery.” From 2000 to 2024, the top 12 keywords with strong citation bursts in post-stroke nutrition research each have designated “start” and “end” years, marking their active periods in academic citations, with red indicating a sudden surge in citations during specific periods. “Blood pressure” (2000–2013) and “cardiovascular diseases” (2001–2018) had relatively long periods of citation bursts, while “recovery” (2019–2021) and “management” (2017–2022) reflect recent research trends and focus. Changes in keywords and timing of citation bursts illustrate shifts in research focus, from “nutritional screening surveys” (2003–2004) to recent focuses on “recovery” and “management,” likely reflecting increased attention to disease management and patient rehabilitation processes. Figure 12 shows the cross-links in impact graphs among the top 20 countries, institutions, and keywords, clearly indicating that the 20 most co-cited keywords are closely related to post-stroke nutrition research.

**Table 6:** Keyword frequency.

Rank	Keyword	Occurrences
1	Stroke	243
2	Nutrition	130
3	Malnutrition	106
4	Ischemic stroke	88
5	Risk	86
6	Mortality	80
7	Dysphagia	68
8	Rehabilitation	59
9	Enteral nutrition	56
10	Cardiovascular-disease	55
11	Prevalence	51
12	Outcome	50
13	Coronary heart disease	44
14	Diet	44
15	Risk factor	44
16	Meta-analysis	38
17	Prevention	38
18	Disease	36
19	Health	33
20	Blood pressure	32

## 3 Discussion

### 3.1 Geographic analysis of publications

Among the top 10 countries/regions and institutions, the majority are from Europe, North America, and Asia. This implies that, aside from China and India, most are developed nations. This pattern establishes a clear correlation between academic output and economic strength, indicating that developed nations or advanced institutions have significant advantages and influence in this field, potentially reflected in higher prevalence rates, better funding support, or more advanced medical research facilities. Visual maps show how different regions interact and collaborate in stroke research, with particularly notable interactions between North America, Europe, and Asia, indicating a global effort to address stroke rehabilitation issues, with significant contributions from these countries/regions. Notably, China has become the country with the highest academic output. At that time, China was recognized as an emerging region in post-stroke nutrition research, a shift that highlights China’s rapid progress and significant leaps in this field. The US surpasses China in h-index and average citations per paper (AC/P), reflecting its dominance in both original article output and research collaboration. This gap underscores the importance of publications in terms of both quantity and quality, as well as their overall impact. Several factors contribute to the current distribution of outcomes. Stroke is a major public health issue in the United States, leading to numerous deaths and significant disabilities each year. The high prevalence of stroke has led the US government to invest heavily in research related to medical fields. The United States’ strategic goal in global health is to lead in medical and health research. By conducting in-depth research on significant health issues such as stroke, the US not only improves the health of its citizens but also plays a leading role in global health affairs. In summary, data analysis underscores the importance of international cooperation in advancing research in post-stroke nutritional interventions. Institutions and countries with high output not only lead in innovation but also play a crucial role in shaping the global research agenda. The geographic distribution of research activities indicates that, while the United States and China lead, global participation is crucial for the diverse application of research findings and the exchange of ideas worldwide.

### 3.2 Summary of research hotspots

From 2000 to 2003, the research focus primarily centered on nutritional supplements, particularly the critical roles of dietary potassium, folic acid, vitamin B6, and vitamin B12, such as reducing inflammatory markers like C-reactive protein (CRP) and homocysteine. Researchers have repeatedly focused on the active ingredients in nutritional supplements and explored their significance in reducing the risk of stroke. A significant inverse, dose-dependent relationship exists between the inflammatory marker C-reactive protein (CRP) and B vitamins. Increased folic acid intake significantly reduces the risk of ischemic stroke in men but shows no significant effect on hemorrhagic stroke. Further research has confirmed that breakfast cereals rich in folic acid, vitamin B6, and vitamin B12 effectively increase levels of B vitamins in the body and reduce concentrations of homocysteine, thereby helping to reduce the risk of stroke (37–40). This finding laid the foundation for the subsequent application of nutritional science in stroke patients, guiding researchers in exploring the protective effects of these nutrients and their impact on cerebrovascular health.

From 2004 to 2005, the number of studies significantly increased for the first time, with research hotspots focusing on the assessment of nutritional status in stroke patients and its relationship with their biomarkers. For example, studies on antioxidants. Researchers of that era displayed extraordinary foresight, systematically identifying areas of study in post-stroke nutrition related to post-stroke feeding methods, the relationship between trace elements in dietary intake and stroke, nutritional status indicators at the time of hospital admission for stroke patients, and changes in food or nutrient intake related to biomarkers. Wang Y and others, through foundational research with the middle cerebral artery occlusion (MCAO) rat model, discovered that antioxidants found in blueberries, spinach, and spirulina could effectively reduce the production of free radicals and neurodegenerative changes, thereby decreasing neurological damage caused by cerebral ischemia. Therefore, long-term consumption of foods rich in antioxidants such as blueberries, spinach, and spirulina can significantly alleviate brain damage in a rat model of ischemic stroke and enhance post-stroke motor activity. Khanna S and others found that natural vitamin E (*α*-tocotrienol) has significant neuroprotective properties and can effectively reduce damage caused by cerebral ischemia. Thus, α-tocotrienol could be considered a valuable therapeutic agent. Furthermore, the activity of caspase-3 is significantly reduced, indicating decreased cellular apoptosis. These findings emphasize the direct role of specific nutrients in mitigating neurological damage after a stroke, providing a scientific basis for nutritional therapy strategies in stroke rehabilitation. These studies encouraged subsequent research to focus more on the reparative effects of specific nutrients on neurological damage.

In 2006, significant breakthroughs were made in research on folic acid fortification policies, providing important references for the evaluation of the effects of subsequent large-scale nutritional policy interventions. A 2006 study published in the high-impact journal “Improvement in Stroke Mortality in Canada and the United States, 1990 to 2002” systematically assessed changes in stroke mortality rates in Canada and the United States from 1990 to 2002. The study utilized baseline population data from the Centers for Disease Control and Prevention (CDC) and Statistics Canada to analyze stroke mortality rates before and after the implementation of folic acid fortification policies. The results indicated that folic acid fortification policies significantly reduced stroke mortality rates in both countries, correlating with a significant reduction in homocysteine levels in stroke patients, thereby confirming the strong link between blood homocysteine levels and stroke risk. These findings emphasized the effectiveness of public health policies in reducing stroke mortality and highlighted the crucial role of nutritional interventions in managing cerebrovascular disease risk.

The 2009 study titled “Mediterranean Diet and Incidence of and Mortality from Coronary Heart Disease and Stroke in Women” conducted a long-term follow-up on female nurses aged 38 to 63 without a history of cardiovascular disease or diabetes, exploring the relationship between the Mediterranean diet and stroke incidence and mortality. The study utilized a food frequency questionnaire to gather dietary data and calculate the Mediterranean diet score (aMED), which accounted for high intake of vegetables, fruits, nuts, whole grains, fish, legumes, and monounsaturated fats, low intake of red and processed meats, and moderate alcohol consumption. After adjusting for various confounding factors, the study found that a higher Mediterranean diet score significantly reduced stroke incidence by 13% and mortality by 18%. The results confirmed the benefits of the Mediterranean diet in preventing stroke and provided a scientific basis for developing diet-based public health strategies.

From 2010 to 2023, there was a steady annual increase in the number of publications, indicating that the field of post-stroke nutritional medicine has entered a phase of steady development.

From 2010 to 2014, studies demonstrated the relationship between nutrients (such as vitamin D and B vitamins) and ischemic stroke. Notably, vitamin D deficiency was independently associated with poor outcomes in stroke patients. These findings provided empirical evidence for the role of nutrients in neuroprotection, and sparked subsequent research into the role of additional micronutrients in stroke prevention and recovery.

A pivotal 2012 study focused on the relationship between various dietary protein sources and stroke risk. Through long-term follow-up and systematic assessment of major protein sources, including red meat, poultry, fish, dairy, and nuts, the study revealed significant differences in stroke risk associated with different protein sources. The results indicated that high red meat intake was linked to an increased stroke risk, while high intake of fish and nuts was linked to a reduced stroke risk. Additionally, the study identified gender differences in the relationship between protein sources and stroke risk. These findings offer valuable insights for public health policy formulation, emphasizing the need to consider dietary protein sources in stroke prevention strategies.

In 2014, researchers highlighted the relationship between serum 25-hydroxyvitamin D levels and ischemic stroke, including its subtypes, and their association with adult homocysteine levels. The study demonstrated that vitamin D deficiency is independently associated with ischemic stroke, especially when serum 25-hydroxyvitamin D levels fall below 21 ng/mL, where it negatively correlates with homocysteine levels. Additionally, folic acid, as a cofactor in one-carbon metabolism, can reduce the neurotoxicity induced by high homocysteine levels, thereby protecting neuronal integrity, although some clinical trials and meta-analyses suggest that the effects of folic acid supplementation are controversial. In the same year, researchers investigated the nutritional status of stroke patients based on feeding methods and evaluated the impact of specific tools in assessing the nutritional status of patients at risk of stroke (40–42). The main risk factors for nutritional deficiency in stroke patients include dysphagia, cognitive impairments, and variations in feeding methods; therefore, regular nutritional assessments are crucial for early detection of malnutrition and timely intervention.

From 2015 to the present, there has been a noticeable growth in the volume of published literature, especially in the diversification of research directions within the field of post-stroke nutrition. After 2015, the diversity and complexity of research related to stroke nutrition further increased, with nutritional assessment tools and support strategies becoming focal points of investigation.

In 2015, research focused on tools for screening and assessing nutritional status (42), secondary prevention through nutrition and dietary adjustments (43, 115), enteral nutrition (44–47), resting energy expenditure (47–49), dietary glycemic load (50), and patients with subarachnoid hemorrhage (51, 52) examining trends in anthropometric nutritional indicators and the effects of obesity, inflammation, and negative nitrogen balance on the nutritional status and outcomes following subarachnoid hemorrhage. The studies revealed that many of the current nutritional screening and assessment tools are unvalidated, highlighting an urgent need to improve and validate these tools for more accurately identifying the risk of malnutrition after stroke. Furthermore, evidence-based guidelines released by the Royal College of Physicians provided practical guidance on nutrients and dietary adjustments for preventing stroke recurrence, indicating that nutritional interventions play a key role in long-term stroke management. Additionally, research on enteral nutrition emphasized the timing of initiation, dosage, and its impact on the prognosis of severe stroke patients, particularly in avoiding complications such as reflux and aspiration. Research indicated that even acute stroke patients should begin appropriate nutritional support soon after admission to avoid the risks associated with malnutrition. Research during this period clarified the critical role of nutritional screening tools in the early detection of malnutrition and the prevention of complications, thereby promoting the application of nutritional management strategies in clinical practice.

From 2016 to 2018, research focused on the positive effects of specific nutritional supplements (such as alpha-linolenic acid and vitamin E) and dietary patterns (like the Mediterranean diet) on stroke rehabilitation. Studies during this period reinforced the significance of nutritional interventions in stroke recovery, and the antioxidant, anti-inflammatory, and neuroprotective effects of these nutrients had a profound impact on subsequent research.

In 2016, research in the field of stroke nutrition further deepened on various types of dietary supplements, such as GrandFusion, alpha-linolenic acid (ALA), serum 25-hydroxyvitamin D, and long-chain omega-3 lipid emulsions. These studies indicate that nutritional supplements with antioxidant, anti-inflammatory, and neuroprotective properties have potential benefits for stroke prevention and rehabilitation. Dietary habits are related to the risk of stroke (53, 54). A Spanish cohort study, with an average follow-up of 13.8 years, found no association between intake of fish, unprocessed red meat, and processed meats and the risk of stroke, even after adjusting for multiple variables (55). This may indicate that the intake of fish and red meat may not be independent determinants of stroke risk, suggesting that a more comprehensive assessment involving other dietary factors and lifestyle might be required in the future. Therefore, assessing stroke risk requires consideration of a broader range of dietary and lifestyle factors. Additionally, this year’s most cited study, titled “Risk of Malnutrition Is an Independent Predictor of Mortality, Length of Hospital Stay, and Hospitalization Costs in Stroke Patients,” demonstrates that malnutrition is an independent predictor of mortality, length of hospital stay, and hospitalization costs in stroke patients. This underscores the importance of nutritional screening and timely intervention in improving stroke patient outcomes. In the same year, research also found that improvements in nutritional status and higher energy intake at the time of admission were significantly related to the recovery of patients’ abilities to perform activities of daily living (ADLs). Specifically, patients receiving nutritional support interventions showed notable improvements in weight gain and functional recovery, and this was particularly relevant to the recovery of daily living activities in elderly stroke patients with nutritional deficiencies during the recovery phase (66).

Research in 2017 further deepened the understanding of nutritional factors in stroke prevention and management. Bonaccio and colleagues’ research revealed an association between fish intake in the Mediterranean diet and reduced stroke risk (116). Additionally, the research showed that a malnutrition risk score is an effective tool for predicting whether elderly stroke patients can resume a normal diet. Vitamin D research further confirmed its deficiency is linked to the severity and poor prognosis of ischemic stroke, particularly evidenced among the Chinese population in Hong Kong. Simultaneously, a significant study published in JAMA assessed the relationship between specific dietary factors and cerebrovascular disease mortality using NHANES data, emphasizing the importance of appropriate nutritional intake in disease prevention (12). Additionally, Patejdl and colleagues noted that gastrointestinal dysfunction is common among stroke patients, calling for increased attention to nutritional absorption and digestive system function during stroke rehabilitation. These studies collectively emphasize the importance of considering nutrition and digestive health in the prevention and treatment of stroke, providing guidance for clinical practice and raising public awareness about the importance of a healthy diet (55).

In 2018, a multidisciplinary team developed the ESPEN guidelines on optimal nutritional treatment for patients with neurological diseases (117), marking a significant advancement in nutritional management strategies for stroke patients. The guidelines comprehensively cover nutritional assessments, interventions, and care plans, with a particular emphasis on thorough nutritional risk screening at admission. They provide detailed assessment and intervention recommendations for patients identified as at nutritional risk. Specific measures include the use of oral nutritional supplements for patients who can eat but have insufficient intake, and the timely initiation of enteral or parenteral nutrition support for those unable to eat orally. Additionally, that year’s research also emphasized the importance of maintaining appropriate protein and fluid intake for stroke patients and provided personalized nutrition plans for patients with swallowing difficulties and gastrointestinal dysfunction (56, 57, 117). These strategies play a crucial role in preventing complications and promoting rehabilitation. Lastly, research has shown that malnutrition in patients with acute ischemic stroke is an independent risk factor affecting prognosis; by adjusting nutritional strategies, significant improvements can be made in patients’ functional recovery and long-term health outcomes. These research findings further confirm the importance of systematically applying scientific nutritional interventions in stroke management.

From 2019 to 2023, there was a rapid increase in publication volume, signifying a fast growth period in the field. Research during this period shifted focus to the long-term effects of malnutrition and its association with dysbiosis of the gut microbiome. These findings offer new directions for future personalized nutritional intervention strategies, highlighting the importance of promoting recovery in stroke patients by improving gut health.

In 2019, research in the field of stroke nutrition focused on understanding the complex relationship between malnutrition and stroke risk. Meta-analyses revealed multiple risk factors associated with malnutrition, such as dysphagia and a history of previous strokes, making it essential to identify these factors early to improve patient outcomes (58). Additionally, studies highlighted the potential impact of proper dietary habits on stroke risk, particularly through investigating how the gut microbiome affects disease risk, to explore the relationship between enteral nutrition and stroke (59–62). Specifically, the promotion of the MIND diet underscored the benefits of combining Mediterranean and DASH diets, particularly in preventing post-stroke cognitive decline (63–65). Concurrently, studies also showed the positive effects of nutritional supplements like pomegranate polyphenols in enhancing cognitive function recovery after stroke. Additionally, research on sarcopenia post-stroke indicated that amino acid supplements rich in leucine could significantly improve muscle mass and function, highlighting the potential of targeted nutritional interventions in improving stroke patient rehabilitation (66). These findings not only provide new perspectives for clinical nutrition support but also lay a solid foundation for future research directions, indicating that there is still tremendous potential in the field of nutritional interventions for stroke, necessitating further research and collaboration to advance the study process.

The years 2020–2022 particularly emphasized the relationship between malnutrition and outcomes after a stroke. Using bioelectrical impedance analysis (BIA) (67, 68) and various nutritional scoring systems such as NRS-2002, CONUT, and PNI (69–71), research highlighted the predictive role of malnutrition in mortality, hospital stay, and healthcare costs among stroke patients. Additionally, nutritional research has also shown the positive impact of nutritional support on muscle mass and functional recovery in stroke patients, revealing a direct link between insufficient energy intake and muscle loss (68, 72, 73) Recent research has also explored the role and impact of the gut microbiome in stroke risk (74) particularly the potential contribution of gut-produced toxic metabolites such as TMAO to stroke risk, emphasizing the importance of maintaining a healthy gut microbiome balance (75) These advances mark an important step towards more refined and personalized medicine, highlighting the central role of nutritional therapy in reducing the burden of stroke and improving patient quality of life.

Studies in 2023 have significantly advanced our understanding of the link between gut microbiome dysbiosis and stroke. Through precise ecological and functional analyses, the research revealed significant changes in the gut microbiota composition of stroke patients, particularly noting increases in Parabacteroides and Escherichia_Shigella and decreases in Prevotella and Faecalibacterium. These changes reflect taxonomic and functional dysregulation in the microbial community, and indicate a potential state of malnutrition (85). Additionally, functional predictions indicated differences in the expression of specific biomarkers in stroke patients, such as increased transposases and peptide/nickel transport system substrate-binding proteins, and decreased RNA polymerase sigma-70 factor and methyl-accepting chemotaxis proteins. These findings highlight that functional imbalances in gut microbiota after a stroke may directly affect nutrient absorption and metabolic status. The research further emphasized the importance of modulating the gut microbiota through nutritional intervention, demonstrating that optimizing nutritional support can not only improve the health of the gut microbiota but also significantly reduce the disability and mortality rates of stroke patients. These findings provide a scientific basis for targeted nutritional and microbial interventions in clinical settings for stroke patients, promoting the application of personalized medicine in stroke management, enhancing the role of gut health in comprehensive treatment strategies, and paving new directions for future stroke prevention and treatment.

## 4 Research hotspots

Using reference and keyword clustering methods, research hotspots in post-stroke nutrition are focused on four areas: risk of malnutrition, nutrients, nutritional management of patients hospitalized for acute stroke rehabilitation, and all-cause mortality.

### 4.1 Nutritional deficiency

According to ESPEN, malnutrition is a condition resulting from insufficient nutritional intake or absorption disorders, characterized by changes in body composition and a decline in cell mass, which subsequently affects physical and mental functions and leads to the deterioration of clinical outcomes in diseases (120). Malnutrition in stroke patients can lead to poor outcomes and is influenced by factors across multiple dimensions (58) such as biological aspects: age, gender, severity of illness; clinical aspects: dysphagia, gastrointestinal dysfunction, chronic diseases (diabetes, hypertension, etc.); socio-economic aspects: economic status, family and community support, smoking, mood, feeding methods, etc. (86–88, 121). Understanding the extent to which these factors contribute to post-stroke malnutrition is crucial. Previous studies have found statistically significant associations between malnutrition upon admission, dysphagia, diabetes, tube feeding, and reduced consciousness levels with malnutrition (89). Patients malnourished at the time of admission already lack essential nutrients needed for brain cell repair and bodily function maintenance. Malnutrition can weaken the immune system, making patients more susceptible to pneumonia, potentially extending hospital stays or even resulting in death. Dysphagia affects the intake of food and liquids in stroke patients, significantly impacting their quality of life. Diabetes can affect the recovery process and prognosis after a stroke, where restricting certain foods leads to nutritional imbalances, and metabolic disorders reduce the efficiency of protein and other nutrient utilization. Tube feeding can be beneficial for reducing the risk of malnutrition in stroke patients, but improper nutritional formulations, insufficient feeding volumes, or poor absorption by the patient can also lead to malnutrition. Patients with reduced levels of consciousness often cannot communicate their needs and discomforts and may be unable to eat independently. Previous research has found a strong correlation between older age and malnutrition, with the risk of malnutrition significantly increasing with age. However, due to the diversity of past study designs and the difficulty in data aggregation, conducting comparative studies across different age groups and long-term longitudinal tracking of stroke patients by age group to observe the impact of age on malnutrition in stroke patients is a future research trend. Although previous studies have extensively investigated post-stroke risk factors, the patient populations in most studies vary in geography, ethnicity, and socio-economic status, potentially affecting the universality and relevance of identified risk factors. Studies on risk factors show significant heterogeneity, with many failing to adequately adjust for all relevant confounding variables. Future research designs should more comprehensively consider and control for potential confounders, such as lifestyle and psychosocial factors, using refined data collection and analysis to better isolate independent factors of malnutrition (69, 122).

### 4.2 Nutrients

The relationship between B vitamins and stroke primarily involves three aspects: homocysteine metabolism, brain health maintenance, and protective effects. Vitamins B6, B12, and folate, well-documented in dietary supplements, play crucial roles in stroke prevention and management. Accumulation of homocysteine in the blood increases the risk of arteriosclerosis and blood clot formation. Vitamins B6 and B12 lower homocysteine levels by participating in its metabolism, thereby reducing the risk of thrombosis. Additionally, vitamins B6 and B12 have antioxidant effects that reduce oxidative damage and tissue inflammation in stroke patients. Low vitamin B12 levels can predict the risk of ischemic stroke, and deficiency may lead to more severe coordination and balance impairments, including fatigue and depression, post-stroke. Early vitamin B12 supplementation can improve the short-term prognosis of ischemic stroke patients, reduce neuronal apoptosis, and possibly aid in brain neuron remodeling. Furthermore, vitamin B12 benefits endothelial cell function, enhances vascular elasticity and stability, reduces the risk of atherosclerosis and thrombosis, and lowers stroke incidence. B vitamins aid in neurotransmitter synthesis and myelin sheath maintenance, positively impacting brain health and functional recovery. Research indicates that one-carbon supplements, including 5-methyl THF, vitamin B12, and choline, play a key role in supporting recovery after ischemic stroke. Moreover, both homocysteine and vitamin B6 are significantly linked to the risk of premature ischemic stroke, although they are not directly related to each other (86, 90–94, 123–124).

Nitrogen balance is a key indicator for assessing the overall nutritional status and protein metabolism in stroke patients, representing the difference between nitrogen intake and output in the body. After a stroke, the inflammatory response in patients intensifies, leading to increased protein breakdown. Without adequate nutritional support, this can result in negative nitrogen balance, or muscle loss due to reduced activity and bed rest, further affecting nitrogen balance. In the nutritional management of stroke patients, nitrogen balance can help determine whether the nutritional support is sufficient or if there is ongoing muscle wasting or other forms of tissue breakdown. When malnutrition occurs, the amount of nitrogen excreted exceeds intake, resulting in a negative nitrogen balance (51, 57). Evidence suggests that nitrogen balance is used to assess nutritional support in stroke patients. Studies show that early enteral nutrition significantly improves nitrogen balance and reduces the risk of stroke-related complications, such as dysphagia and malnutrition.

### 4.3 Nutritional management of inpatient rehabilitation for acute stroke

Nutritional management for patients rehabilitating from stroke is a complex and comprehensive topic, currently encompassing a wide range of research including nutritional assessment (95), dietary planning (96), nutritional care practices (97), management of dysphagia, and prevention of complications (98) Key emphases include the impact of nutritional status on rehabilitation, randomized controlled trials on nutritional supplementation, and systematic nutritional assessment and management. Researchers like Miller, C have used cross-sectional surveys to compare current in-hospital hydration and nutritional care experiments for stroke patients in the UK and Australia, finding that dehydration in post-stroke patients leads to adverse outcomes, including increased mortality. There is a need to enhance the implementation and use of evidence-based protocols in stroke hydration and nutritional care, and to develop standardized assessment tools to improve patient outcomes (99). Additionally, high energy intake is associated with the rehabilitation practices of patients with sarcopenia.

The Comprehensive Dietary Antioxidant Index (CDAI) serves as a nutritional tool (100) to assess the overall antioxidant capacity of an individual’s daily food consumption. Individuals with a higher CDAI, indicating a diet rich in various antioxidants, have a relatively lower risk of stroke. Professional organizations and government agencies recommend weight management for overweight or obese patients with acute ischemic stroke (101). However, weight loss methods have not been thoroughly tested in stroke patients. Large-scale trials on the feasibility and safety of partial meal replacement (PMR) interventions for weight loss are currently underway. Based on current research progress, future studies should enhance outcome measurement, expand sample sizes, and extend follow-up periods to evaluate the impact of partial meal replacement diets on long-term weight maintenance and overall health post-stroke. In summary, effective post-stroke nutritional risk management necessitates systematic assessment and intervention. Comprehensive management, including nutritional assessment, dietary planning, nutritional care practices, dysphagia management, and complication prevention, is essential for improving patients’ nutritional status and enhancing quality of life and rehabilitation outcomes for stroke patients.

### 4.4 Stroke and all-cause mortality

Previous studies have found that dietary antioxidant intake (including vitamins A, C, E, zinc, selenium, and carotenoids), dietary magnesium intake, and serum levels of albumin and globulin are negatively correlated with stroke mortality risk. Antioxidant vitamins A, C, and E intake is negatively correlated with the risk of post-stroke depression in the general population, though there is controversy over whether dietary carotenoids can similarly reduce depression risk (102, 103). Dietary magnesium in the human body regulates glucose metabolism, protein synthesis, and muscle and nerve functions. About one-third of stroke patients have insufficient magnesium intake. Research has indicated a negative correlation between dietary magnesium intake and stroke mortality, suggesting that the beneficial effects of high magnesium intake may be due to its neuroprotective properties. It has also been noted that total calcium, sodium, and potassium intake are not related to stroke mortality. The serum albumin/globulin ratio (A/G) is an indicator of inflammation and nutritional status, with abnormalities in serum A/G observable in malnutrition. Studies have shown that relatively higher serum A/G levels can reduce the risk of adverse outcomes in patients with acute ischemic stroke. However, the specific appropriate dosage of albumin to reduce stroke risk requires longer follow-up trials for verification.

## 5 Future research trends

The use of CiteSpace software for keyword grouping highlights future trends in post-stroke nutrition in four key areas: functional recovery and nutritional interventions, risk assessment of malnutrition, the relationship between gut microbiota and stroke, and the role of alpha-linolenic acid.

### 5.1 Functional recovery and nutritional intervention

Nutritional intervention has been shown to be an essential component of medical nutrition therapy, as noted by ESPEN 2018 (24) this approach aims to help patients meet their energy requirements, prevent weight and fat loss, and avert further catabolism. This involves tailoring nutritional interventions and dietary prescriptions based on the individual characteristics of stroke patients (such as physiological status, health condition, lifestyle, nutritional status, etc.), and continuously monitoring the patient’s nutritional intake and status to develop nutritional intervention plans, as the nutritional needs of stroke patients vary according to their rehabilitation stage, complications, and long-term health goals (125). A comprehensive assessment of health and nutritional status is necessary, including patient weight, body composition (muscle mass, fat ratio, protein requirements, energy, etc.), laboratory indicators (blood glucose, cholesterol, serum albumin, C-reactive protein levels), and methods of food administration (enteral, parenteral, oral, intravenous). Utilizing genetic testing and biomarkers to identify patients’ metabolic capacities and sensitivities to specific nutrients, this approach checks for genetic variations that may affect individual needs for vitamin C, folate, or fatty acids. Stroke patients may require specific nutrients to support neural recovery and reduce inflammation, such as branched-chain amino acid (BCAA) supplements to alleviate stroke-induced muscle loss, and pomegranate supplements to improve cognitive impairments following ischemic stroke, omega-3 fatty acids, antioxidants (vitamin E and selenium), B vitamins, and potassium are considered. Additionally, patients’ dietary preferences, cultural backgrounds, and lifestyles (eating habits, work, and activity levels) are taken into account to ensure that the nutrition plan is sustainable and acceptable. In improving post-stroke cognitive function, the Mediterranean-DASH Intervention for Neurodegenerative Delay (MIND) diet has proven effective and promising. This diet combines the benefits of both the Mediterranean and DASH diets, specifically emphasizing brain-beneficial foods including whole grains, green leafy vegetables, berries, and nuts, while restricting red meat, cheese, and fried foods. In a community cohort study involving 106 post-stroke patients, it was found that those adhering to the MIND diet experienced a slower rate of cognitive decline during the follow-up period. The MIND diet may improve cerebrovascular health by providing anti-inflammatory and antioxidant nutrients (64). The intake of leucine amino acid supplements and high-energy nutritional supplements significantly benefits the rehabilitation of muscle function and enhancement of daily activity capabilities in stroke patients. Leucine is a critical trigger for muscle protein synthesis, essential for preventing muscle loss. Supplementing with leucine in the diets of stroke patients aids in increasing muscle mass, alleviating muscle atrophy, and enhancing muscle strength, which assists in restoring motor functions and improving independence in daily activities like walking and stair climbing. Moreover, high energy intake is essential for effective rehabilitation, enabling patients to participate in more frequent and intensive physical rehabilitation exercises. Nutritional interventions should be regularly monitored and adjusted based on patient progress and feedback, including adjustments to energy and protein intake and routine calculation of targeted calories or specific nutrient supplements. There is a need to develop more targeted nutrition and rehabilitation strategies in the future to enhance muscle function and overall recovery for stroke patients. Researchers are tasked with exploring different nutritional supplementation approaches for stroke patients, using standardized assessment tools, planning diets appropriately, and considering patients’ specific dietary needs and preferences to develop and implement systematic nutritional evaluation and management strategies.

### 5.2 Assessment of malnutrition

With increasing recognition of malnutrition risks following a stroke, there is a growing need for nutritional screening tools to assess malnutrition more accurately. Tools for assessing malnutrition can pinpoint malnutrition risks in stroke patients, enabling healthcare teams to identify at-risk individuals and promptly administer nutritional support, which can significantly enhance patients’ functional outcomes. Tools for assessing malnutrition are extensively utilized in clinical practice. The Nutritional Risk Screening (NRS-2002) tool, incorporating both initial screening and comprehensive evaluation, considers the severity of illness and nutritional status in determining nutritional risk. In clinical practice, performing these assessments requires specific expertise and training from evaluators, and some aspects of the scoring may be subject to subjective interpretation. The CONUT (Control of Nutritional Status) score uses laboratory parameters such as serum albumin, total cholesterol, and lymphocyte count for quick assessments, minimizing subjective bias from evaluators. However, its reliance on biochemical indicators may neglect clinical symptoms, making it potentially less comprehensive than other assessment tools. The Geriatric Nutritional Risk Index (GNRI) takes into account the physiological characteristics of the elderly, particularly muscle mass and body weight. Improvements in nutritional status, especially in the GNRI, can effectively enhance Functional Independence Measure (FIM) scores and accelerate patient rehabilitation. Low GNRI values are significantly associated with persistent swallowing disorders (PSD), especially patients with moderate or severe malnutrition are more likely to experience persistent PSD compared to those without nutritional risks (104). This finding highlights the importance of conducting nutritional assessments at admission to identify patients at risk of persistent swallowing disorders (32, 105, 126, 127). Research has found that a lower GNRI in elderly patients following acute ischemic stroke is independently associated with the development of post-stroke cognitive impairment (PSCI), suggesting that nutritional status may influence cognitive recovery after a stroke, particularly in overall cognitive and executive functions. In summary, the accuracy of GNRI assessments may be compromised if the information on weight and height is inaccurate, and it does not comprehensively consider all aspects of a patient’s health. Nutritional assessment tools have shown significant clinical value, and each tool has its merits. While existing tools have provided important clinical assistance, there is still a need for further improvement and development. Some of the existing tools do not specifically target certain populations, such as patients with specific underlying diseases, and there is a lack of standardization among different assessment tools. The application and interpretation of existing nutritional assessment tools vary across different medical institutions and studies. The standardization of future assessment tools needs further research to ensure that their quality and reliability meet international standards. Many assessment tools primarily focus on the short-term risk of malnutrition, with insufficient monitoring and assessment of long-term nutritional status, potentially affecting chronic disease management and long-term health planning. This issue needs to be addressed in the future to provide timely feedback on the effectiveness of nutritional interventions.

### 5.3 Enteral nutrition

Enteral nutrition aids patients who have intact gastrointestinal function but are unable to eat normally by providing essential nutrients. Nutrients are delivered continuously or intermittently through nasogastric tubes, nasoenteric tubes, gastrostomies, or enterostomies, including proteins, fats, carbohydrates, vitamins, and minerals. Compared to parenteral nutrition, enteral nutrition better maintains gastrointestinal function, enhances systemic immune function and biological defense capabilities, and reduces the risk of infections (106–109). It lowers the incidence of pneumonia and sepsis, promotes nutritional absorption, maintains nutritional status, accelerates recovery, and improves quality of life (110). Studies have shown that combining enteral nutrition with gut microbiota can improve the nutritional status of acute stroke patients, reduce the incidence of infection complications and gastrointestinal motility disorders, lower the risk of SAP in acute IS patients, and decrease ICU/hospitalization time (111). Pneumonia, particularly aspiration pneumonia, is a common clinical issue associated with a high incidence in enteral nutrition. Improper handling of nasogastric or gastrostomy procedures can cause nutritional fluid to reflux into the esophagus and be mistakenly aspirated into the trachea. Prolonged enteral nutrition may alter the patient’s gut microbiota, which can affect intestinal barrier function and indirectly increase the risk of infections (62). Future research needs to precisely consider the impact of aspiration pneumonia on patients and develop new technologies for real-time monitoring of gastric content reflux. Current clinical research has found that low-calorie enteral nutrition (40–60% of estimated caloric needs), full-calorie enteral nutrition (70–100% of estimated calories), and modified full enteral nutrition (full-calorie with prokinetics) are associated with poor stroke outcomes and increased mortality (112). However, larger sample sizes are needed to further confirm the effects of modified full enteral nutrition or other enteral nutrition schemes, such as varying nutritional formulas, dosing rates, and timing, to guide clinical practice.

### 5.4 *α*-Linolenic acid supplements

Alpha-linolenic acid (ALA) as a nutritional supplement is attracting growing interest for its application in stroke management. Alpha-linolenic acid acts as a plant-derived precursor to long-chain omega-3 fatty acids (EPA and DHA), requiring sufficient dietary intake for the body to synthesize these essential fatty acids. Alpha-linolenic acid may have a pre-conditioning effect on the brain, increasing brain tissue’s resistance to ischemic events. This preconditioning effect is mediated by activating specific signaling pathways, which enhance cellular tolerance and thus mitigate the damage caused by subsequent severe ischemia. Studies have shown that alpha-linolenic acid can enhance the tolerance of brain cells, reduce damage caused by ischemic reperfusion in brain tissue, promote cerebral vasodilation and neuroplasticity, reduce inflammation in brain cells, improve vascular health, and potentially enhance cerebral blood flow and neuroprotection by acting directly on blood vessels and neurons (113). Fundamental research has shown that alpha-linolenic acid supplements enhance the survival of hippocampal neurons, thus improving spatial learning and memory in mice post-stroke (114). Therefore, incorporating alpha-linolenic acid into the daily diet, such as through flaxseed oil, walnuts, and certain green leafy vegetables, can have a positive effect on cerebrovascular health. Our series of studies on alpha-linolenic acid reveal that monitoring serum biomarkers, adjusting intake, and researching its anti-inflammatory and neuroprotective mechanisms offer insights into how it affects stroke recovery, providing a clear research trajectory for the future. This approach involves not only focusing on the nutrient itself but also optimizing its use and monitoring its activity in the body through biomarkers.

## 6 Limitation

1. Our search did not include other academic databases, which may have resulted in the omission of some influential literature.
2. Secondly, although our aim is to analyze global trends in stroke-related nutrition research, the limited range of languages in our study may have introduced selection bias.
3. Our analysis included both articles and reviews, which may introduce bias when assessing the academic impact based on citation counts.
4. This analysis focuses on global research trends, key literature, and keywords related to stroke and nutrition, resulting in limited exploration of specific nutritional interventions in clinical settings. Future research could further integrate quantitative and qualitative methods to explore the application effects of nutritional interventions in real clinical environments. This approach would address the shortcomings of bibliometric studies in focusing on patient-level details.

## 7 Conclusion

According to Figure 2’s publication trend graph, the number of publications has generally shown an annual increase, with the curve peaking in 2022. Additionally, the graph shows that the growth rate of citations surpasses that of publications, with a peak in 2023. In recent years, with the development and popularization of modern technologies such as nutrigenomics and metabolomics, research in this field has significantly deepened, revealing the clinical significance of nutritional interventions in stroke rehabilitation. This emerging research direction has rapidly developed in a short period. However, from 2000 to 2014, progress in research on the specific applications and effects of nutritional interventions in stroke rehabilitation was slow. Since 2015, the field has garnered significant attention from clinicians and researchers, with a rapid increase in related research publications. Research in post-stroke nutrition has become an established direction, with continuous emergence of results from randomized controlled trials, prospective, retrospective, or population-based cohort studies, providing insights for interventions in stroke treatment.

Nishioka Shinta is the most prolific author in this field, having published 11 papers with an H-index of 10. He and his team primarily focus on research concerning malnutrition, muscle loss, malnutrition risk screening, and nutritional assessment in stroke rehabilitation patients. These studies are crucial for improving the rehabilitation process and quality of life for stroke patients, particularly in providing targeted nutritional interventions and enhancing long-term rehabilitation outcomes. Additionally, as shown in Tables 1, 2, China publishes the most in this research field. However, a citation survey reveals that the most cited articles primarily come from leading journals such as “Stroke” and “Clinical Nutrition.” The authors of these journals are primarily non-Chinese, predominantly from the United States, the UK, Canada, Australia, and various European countries. Although most articles related to stroke nutrition come from China, there may be shortcomings in research design, methodological innovation, or international impact of the results, which could be a major reason for their lower representativeness.

Overall, bibliometric results indicate that research in stroke nutrition is still flourishing, with researchers from various countries contributing to different aspects of the field. The scientific output in post-stroke nutrition marks a growing body of evidence supporting nutrition’s key role in enhancing recovery, reducing complications, and improving the quality of life for stroke survivors. This work provides a solid foundation for future research and clinical guidelines, ensuring that nutritional care continues to be a key component of overall stroke rehabilitation.

During the preparation of this work, the author used Chatgpt 4.0 for article touch-ups. After using this tool, the author reviewed and edited the content as needed and took full responsibility for the content of the publication.

## Data Availability

The original contributions presented in the study are included in the article/supplementary material, further inquiries can be directed to the corresponding author.
